# Global expression of noncoding RNome reveals dysregulation of small RNAs in patients with HTLV-1–associated adult T-cell leukemia: a pilot study

**DOI:** 10.1186/s13027-020-00343-2

**Published:** 2021-01-09

**Authors:** Andrezza Nascimento, Daniela Raguer Valadão de Souza, Rodrigo Pessôa, Anna Julia Pietrobon, Youko Nukui, Juliana Pereira, Jorge Casseb, Augusto César Penalva de Oliveira, Paula Loureiro, Alberto José da Silva Duarte, Patricia Bianca Clissa, Sabri Saeed Sanabani

**Affiliations:** 1 Laboratory of Dermatology and Immunodeficiency, Department of Dermatology, Instituto de Medicina Tropical de São Paulo, Faculty of Medicine, University of São Paulo, Av. Dr. Eneas de Carvalho Aguiar, 470 3° andar, São Paulo, 05403 000 Brazil; 2 Department of Hematology, Faculty of Medicine, University of São Paulo, São Paulo, 05403 000 Brazil; 3 Department of Neurology, Emilio Ribas Institute of Infectious Diseases, São Paulo, 01246-900 Brazil; 4 Pernambuco State Center of Hematology and Hemotherapy, Recife, Pernambuco CEP 52011900 Brazil; 5 Immunopathology Laboratory, Butantan Institute, São Paulo, 05503-900 Brazil; 6 Laboratory of Medical Investigation Unit 03, Clinics Hospital, Faculty of Medicine, University of São Paulo, São Paulo, 05403 000 Brazil

**Keywords:** *Small RNA*, *HTLV-1*, *T cell antigen receptor*, *Asymptomatic carriers*, Massively parallel sequencing

## Abstract

**Background:**

Adult T cell lymphoma/leukemia (ATLL) is a peripheral T-cell neoplasm caused by human T-cell lymphotropic virus-1 (HTLV-1). Small RNAs (sRNAs), including microRNAs (miRNAs), play a pivotal role in the initiation and development of hematological malignancies and may represent potential therapeutic target molecules. However, little is known about how these molecules impact the pathogenesis of ATLL. In this study, we aimed to identify sRNA expression signatures associated with ATLL and to investigate their potential implication in the pathophysiology of the disease.

**Methods:**

Small-RNAseq analysis was performed in peripheral blood mononuclear cells from HTLV-1- associated ATLL (*n* = 10) in comparison to asymptomatic carriers (*n* = 8) and healthy controls (*n* = 5). Sequencing was carried out using the Illumina MiSeq platform, and the deregulation of selected miRNAs was validated by real-time PCR. Pathway analyses of most deregulated miRNA were performed and their global profiling was combined with transcriptome data in ATLL.

**Results:**

The sequencing identified specific sRNAs signatures associated with ATLL patients that target pathways relevant in ATLL, such as the transforming growth factor-(βTGF-β), Wnt, p53, apoptosis, and mitogen-activated protein kinase (MAPK) signaling cascades. Network analysis revealed several miRNAs regulating highly connected genes within the ATLL transcriptome. miR-451-3p was the most downregulated miRNA in active patients.

**Conclusions:**

Our findings shed light on the expression of specific sRNAs in HTLV-1 associated ATLL, which may represent promising candidates as biomarkers that help monitor the disease activity.

**Supplementary Information:**

The online version contains supplementary material available at 10.1186/s13027-020-00343-2.

## Background

Human T-lymphotropic virus type I (HTLV-I) is the first discovered human oncogenic retrovirus and was first isolated from a patient with adult T cell leukemia/lymphoma (ATLL) [1]. It has been estimated that 5–10 million individuals that carry the HTLV-I on a global scale [2]. The occurrence of HTLV-1 disease burden is found to be unevenly distributed with some regions disproportionately affected such as South America and southwest Japan [3]. Although most infections with HTLV-I are asymptomatic, in other patients and under some circumstances not completely understood, the virus may cause a variety of symptoms including ATLL and a chronic progressive neuromyelopathy (tropical spastic paraparesis/HTLV-1-associated myelopathy [TSP/. HAM] [4]. ATLL patients initially evolve to a preleukemic phase that is characterized by the accumulation of leukemic cells in peripheral blood circulation, cutaneous disorders, and lack of contribution of other organs [5]. Studies conducted in endemic areas reported that 2.5 to 5% of the asymptomatic HTLV-1 carriers develop ATLL after a long latency period [6, 7]. The reasons why do some people with HTLV-1 get symptoms while others don’t, appear to be complex and probably dependent on factors related to the host and virus, however, the underlying molecular mechanism(s) have not been fully elucidated [8]. Molecular studies have demonstrated that the impairment of multiple cellular functions by viral genes (e.g., *tax* and HTLV-I basic leucine zipper (*HBZ*)), epigenetic alterations in DNA methylation, and the host immune system may promote the leukemogenesis of ATLL [9, 10]. Recent studies have demonstrated the potential role of HBZ in the oncogenesis by inducing the proliferation of HTLV-1 infected cells, upregulating human telomerase reverse transcriptase (hTERT) transcription, suppressing apoptosis, and disrupting host genomic integrity depending on several microRNAs (miRNA) that are HBZ-inducible [11–14].

Small RNAs (sRNAs) including MicroRNAs (miRNAs) are endogenous, ~ 22-nt noncoding RNAs (ncRNAs) that have been implicated as important players in modulating the translation of messenger RNA (mRNA), establishing chromosomal architecture, and providing defense against viruses and mobile genetic elements (transposons) [15–17]. These noncoding molecules bind to the 3′-UTR of target mRNAs, thereby repressing their translation and/or promoting their decay [18]. There are different types of structural ncRNA such as transfer RNA (tRNA) and ribosomal RNA (rRNA), small nuclear RNAs (snRNAs), small nucleolar RNAs (snoRNAs), and small cytoplasmic RNA (scRNA). Regulatory ncRNAs are divided into various subclasses including microRNAs (miRNAs), PIWI-interacting RNAs (piRNAs), and long ncRNAs (lncRNAs) [19]. The miRNAs are perhaps the most intensively studied and arguably the well-understood subclass. They are single-stranded molecules with a length of 18–25 nucleotides (nt) that have been recognized as key regulators of gene expression and play a vital role in regulating various aspects of circadian clock function including those involved in cell proliferation, differentiation, and apoptosis [20, 21]. Mature miRNAs are transcribed by RNA polymerase II as large RNA precursors called pri-miRNAs through sequential cleavages by Drosha and Dicer and are composed of a 5′ cap and poly-A tail [22]. To repress translation and degrade the target mRNAs, miRNAs need to be loaded onto Argonaute protein (AGO protein) to form the RNA-induced silencing complex (RISC) [23]. Several miRNAs have been identified to function in controlling the majority of mRNAs in the human genome [24]; thus they exhibit a variety of crucial regulatory functions in a wide range of biological processes, including tumorigenesis [25], hematopoiesis [26], epigenetics [25], and angiogenesis [27, 28].

Available data from recent studies on miRNA expression profiles in HTLV-1/ATLL cell lines and primary ATL cells have identified many dysregulated miRNAs. For instance, Pichler et al. [29] analyzed the expression of miRNAs using miRNA array technology in HTLV-1–transformed cell lines and demonstrated an upregulation of miR-21, miR-24, miR-146a, and miR-155 and downregulation of miR-223. In a similar study using primary ATL cells, Bellon et al. [30] demonstrated an upregulation of miR-155 and miR-142-3p and down-regulation of miR-181a, miR-132, and miR-125a; nevertheless, the results from a previous study of miRNA precursor expression profiling in HTLV-1–transformed human T-cell lines and primary peripheral blood mononuclear cells (PBMCs) from ATLL patients revealed six miRNAs including miR-93 and miR-130b that were consistently up-regulated [31]. One important finding in that study is that the authors provided solid evidence that the tumor suppressor protein, tumor protein 53-induced nuclear protein 1 (TP53INP1), is a functional target of miR-130b in cell-growth dysregulation of HTLV-1. In another study, Yamagishi et al. [32] used miRNA expression microarray analysis to characterize the miRNA expression signature in the primary ATLL cells. The results from the latter study demonstrated that most of the differentially expressed miRNAs were downregulated, with the most profound suppression detected in miR-31. Interestingly, the same study showed that miR-31 targeted MAP 3 K14 (NIK), which is overexpressed in ATLL cells, leads to constitutive activation of the NF-κB pathway. In this regard, the activation of the NF-κB pathway has been reported to play a crucial role in ATLL pathology [33–35]. Finally, Ruggero and collaborators [36] used deep sequencing technology to determine the profile of miRNAs and tRNA fragments expressed in HTLV-1-infected cells compared to normal CD4+ T cells. The majority of the previously mentioned studies were designed to investigate the expression of specific or diverse miRNAs in cell lines infected with HTLV-1 virus, yet few studies have attempted to identify the expression pattern of sRNAs isolated from human blood samples. Thus, it would be clinically relevant to investigate the global expression of noncoding RNome in clinically well-characterized patients.

In this study, we employed Illumina massive parallel sequencing technology to comprehensively characterize the sRNA expression profiles in the PBMCs of healthy control individuals and HTLV-1 asymptomatic carriers with polyclonal T cell receptor gamma (γ) gene rearrangement, designated here as ASP, and patients with ATLL (acute *n* = 6, chronic *n* = 3, and lymphomatous *n* = 1). The study also aimed to predict target genes and thus provide an additional layer of information that might be useful in understanding host cellular mechanisms as they pertain to promoting or inhibiting virus replication. Our results reveal a set of differentially expressed sRNAs as potential biomarkers to predict ATLL and possibly monitor the dynamics of the disease.

## Materials and methods

### Clinical samples

Peripheral blood samples were collected from healthy donors and patients with their informed consent between 2012 and 2014. The study group included eight ASP patients, ten patients with acute or non-acute ATLL, and five healthy controls (HCs). All ASP patients were diagnosed as HTLV-1 carriers at the time of blood donation. Viral infection was identified by Murex HTLV I + II (Abbott/Murex, Wiesbaden, Germany) and Vironostika HTLV-I/II (bioMérieux bv, Boxtel, Netherlands) HTLV enzyme immunoassays, and infection was confirmed by HTLV BLOT 2.4 (HTLV blot 2.4, Genelabs Diagnostics, Science Park, Singapore). Diagnostic criteria for ATLL included serologic evidence of HTLV-1 infection and cytologically or histologically proven T cell malignancy. ATLL patients were classified according to the Shimoyama criteria into acute, lymphomatous, and chronic types [37].

### Genomic DNA and RNA extraction

Isolation of PBMCs was carried out using a standardized ficoll Hypaque -based protocol and stored in liquid nitrogen until use. Genomic DNA from PBMCs was extracted using the QIAamp blood kit (QIAGEN, Tokyo, Japan) according to the manufacturer’s instructions. The extraction of total RNA and sRNA was conducted using the miRNeasy Mini Kit (Qiagen, Hilden, Germany) in conjunction with the TRIzol (Life Technologies, USA) following the manufacturer’s protocols. The eluted genetic materials were stored at − 80 °C until further use.

### HTLV-1 proviral load determination

The extracted DNA was used as a template to amplify a 97-bp fragment from the HTLV-1 *tax* region using previously published primers [38] and protocol [39]. Amplification and analysis were performed with the Applied Biosystems 7500 real-time PCR system. The standard curves for HTLV-1 *tax* were generated from MT-2 cells of log_10_ dilutions (from 10^5^ to 10^0^ copies). The threshold cycle for each clinical sample was calculated by defining the point at which the fluorescence exceeded a threshold limit. Each sample was assayed in duplicate, and the mean of the two values was considered the copy number of the sample. The HTLV-1 proviral load was calculated as the copy number of HTLV-1 (*tax*) per 1000 cells = (copy number of HTLV-1 *tax*)/(copy number of *RNase P* gene/2) × 1000 cells. The method could detect one copy per 10^3^ PBMCs.

### Analysis of T-cell receptor-γ (TCR-γ) gene rearrangements

A DNA-based polymerase chain reaction (PCR) of rearranged yTCR genes was performed according to the previously described protocol [40]. All patients’ PCR products were analyzed with the 3130 ABI Prism capillary electrophoresis equipment. 0.5 μl ROX, 13 μl Hidi, and 1 μl template DNA sample were added to each well in a 96-well plate. Data were analyzed using Genescan and Genotyper software (Applied Biosystem, Foster City, CA). T cell clonalities were blindly determined by visual examination of the electropherograms by two analysts and further confirmed by an expert hematology pathologist (coauthor JP).

### sRNA construction and massively parallel sequencing (MPS)

For each sample in both groups, sRNA libraries were prepared with the Small RNA v1.5 sample preparation kit as per the manufacturer’s instructions (Illumina, San Diego, CA) and a previously published protocol [41]. Briefly, 5 μl of purified total RNAs were ligated with 1 μl RNA 3′ Adapter and then with a 5′ RNA adapter (Illumina, San Diego, CA). The 5′ adapter also included the sequencing primer. After RT-PCR amplification, the resulting products were analyzed using polyacrylamide gel electrophoresis (PAGE) (6% Novex Tris-borate-EDTA [TBE] PAGE; Invitrogen). After gel electrophoresis, sRNA bands at sizes 145–150 bp were excised and purified. The libraries were normalized to 10 nM and pooled equimolarly in pools of 4 samples per pool. Finally, each pool of libraries was diluted to give a final concentration of 10 pM, then loaded and sequenced on the MiSeq platform (Illumina) with a 36 base single-end protocol, according to the manufacturer’s instructions.

### sRNA data analysis and interpretation

Base-calling, demultiplexing, and trimmed FASTQ files were generated using the MiSeq reporter. Only high-quality reads with a score > 30 on the Sanger scale were considered for further analysis. The reads were aligned against the whole genome build: hg19 using Strand NGS v3.1, which was also used for the analysis of novel molecule discoveries and interpretations. Novel sRNA was discovered and classified by the decision tree method with three-fold validation accuracy using the model previously described by Langenberger et al. [42]. Additionally, only the sRNA sequences that met the minimum read coverage criterion of ≥5 in at least 80% of the samples were considered novel or known sRNA and were included in further analyses. The sRNAs expression MPS data for ASP and ATLL were contrasted against the HC samples to determine differentially expressed sRNAs (analysis of variance [ANOVA] *p-*value of < 0.05 after Benjamini-Hochberg step-up multiple testing correction and fold change of > 2 compared to the HC samples). Heatmaps and/or hierarchal clustering for the significantly differentially regulated sRNA and/or miRNAs were plotted using Strand NGS v3.1.

### qRT-PCR validation of miRNAs

Two miRNAs, namely, hsa-mir-451a (MI0001729) and hsa-mir-144 (MI0000460), which demonstrated a tendency toward strong dysregulation between ATLL vs. ASP vs. healthy samples on MPS analysis were selected and subjected to validation by RT-qPCR. A total of 5 μl of each enriched miRNA was converted into cDNA using the TaqMan™ MicroRNA Reverse transcription kit (REF 4366596, Thermo Fisher Scientific, Inc., Rockford, IL, USA). qRT-PCR was performed using the TaqMan™ Universal master mix II (REF: 4440040, Thermo Fisher Scientific, Inc.) on a 7500 Real-Time PCR system (Thermo Fisher Scientific, Inc.). Each reaction, including no-template negative control, was assayed in triplicate. The relative expression level of miRNA was normalized to the internal control of miR16 (MI0000070). The PCR conditions consisted of UNG activation at 50 °C for 2 min, pre denaturation, and hot start *Taq* activation at 95 °C for 20 s, then 40 cycles of 95 °C for 3 s, and 60 °C for 30 s. For the evaluation of the data, we used the dCT (CT) method (−dCT values = −[CT of target miRNA −CT of internal control miRNA]) [43]. A comparison of the ATLL and ASP groups was performed using the t-test; a *p-*value < 0.05 was considered statistically significant.

### Constructing a regulatory network between miRNAs and their targets

The post-transcriptional gene regulatory network is defined as a directed and bipartite network in which expressions of miRNA-target gene interacting pairs are reversely correlated. The analysis of the network for the interaction of miRNA-messenger RNA (mRNA) putative target was performed using the miRWalk network algorithm.

### Functional annotation and pathway analysis of miRNA target genes

The target genes from differentially expressed miRNAs of ATLL vs. ASP and HC groups were predicted by the miRWalk 3.0 algorithm. After obtaining a list of putative and experimentally validated targets relative to each miRNAs, we further scanned these targets and analyzed them for gene ontology molecular function (GOMF) enrichment terms and Kyoto Encyclopedia of Genes and Genomes (KEGG) pathway classification.

## Results

### Patient characteristics

The characteristics of the subjects in each group are shown in Table 1. All eight participants within the ASP group were female, and the median age was 55 years (range 49, 31–80 years). Five females and five males were assigned to the ATLL group, and the median age of the entire group was 41 years (range 49, 24–73 years). In the leukemic group, six had acute ATLL, three had chronic, and one had lymphomatous types of ATLL. HTLV-1 proviral load levels varied from one copy to 208 copies/10^3^ PBMCs in the ASP group and varied from 104 to 4482 copies/10^3^ PBMCs in the ATLL group (two-tailed *p*-value = 0.02).

**Table 1:** Demographic and clinical characteristics of study subjects

Sample	Sex	Age (Years)	Clinical status	Clonality featuers	Proviral load (copies/1000 PBMCs)	Total input reads	Total filtered reads (%)	Mapped reads
131ASP	Female	52	Asymptomatic	Polyclonal	17	4,029,944	781,412 (19,39%)	3,248,532
146ASP	Female	59	Asymptomatic	Polyclonal	208	12,762,102	2,906,807 (22,78%)	9,855,295
151ASP	Female	72	Asymptomatic	Polyclonal	1	11,798,500	1,847,981 (15,66%)	9,950,519
152ASP	Female	34	Asymptomatic	Polyclonal	60	6,451,236	904,790 (14,03%)	5,546,446
167ASP	Female	58	Asymptomatic	Polyclonal	32	5,947,163	953,127 (16,03%)	4,994,036
172ASP	Female	31	Asymptomatic	Polyclonal	102	4,454,137	737,503 (16,56%)	3,716,634
182ASP	Female	80	Asymptomatic	Polyclonal	22	7,316,342	1,431,104 (19,56%)	5,885,238
188ASP	Female	49	Asymptomatic	Polyclonal	8	9,232,541	2,325,497 (25,19%)	6,907,044
001ATLL	Female	65	Lymphomatous	Monoclona	3297	7,344,758	1,914,409 (26,06%)	5,430,349
005ATLL	Male	73	Chronic	Monoclona	207	5,482,002	1,487,974 (27,14%)	3,994,028
007ATLL	Male	58	Chronic	Monoclona	283	8,320,364	2,056,148 (24,71%)	6,264,216
017ATLL	Female	35	Chronic	Monoclona	104	9,024,187	2,474,763 (27,42%)	6,549,424
171ATLL	Male	56	Acute	Monoclona	957	4,030,123	209,221 (5,19%)	3,820,902
227ATLL	Female	24	Acute	Monoclona	4482	2,473,707	210,428 (8,51%)	2,263,279
238ATLL	Male	34	Acute	Monoclona	2277	2,067,310	187,676 (9,08%)	1,879,634
269ATLL	Male	43	Acute	Monoclona	846	8,771,571	345,333 (3,94%)	8,426,238
275ATLL	Female	34	Acute	Monoclona	508	5,497,660	200,073 (3,64%)	5,297,587
342ATLL	Female	39	Acute	Monoclona	898	6,977,352	750,867 (10,76%)	6,226,485
002HC	Female	38	HC	ND	NA	3,716,097	430,662 (11,59%)	3,285,435
003HC	Male	53	HC	ND	NA	8,962,210	910,815 (10,16%)	8,051,395
004HC	Female	37	HC	ND	NA	5,086,522	845,267 (16,62%)	4,241,255
005HC	Male	32	HC	ND	NA	2,978,098	473,954 (15,91%)	2,504,144
006HC	Female	32	HC	ND	NA	6,700,098	866,374 (12,93%)	5,833,724

*Abbreviations*: *HTLV-1* Human T-lymphotropic virus type I, *ASP* Asymptomatic carriers with polyclonal T cell receptor gamma (γ) gene rearrangement, *ASM* Asymptomatic carriers with monoclonal T cell receptor γ gene rearrangement asymptomatic monoclonal rearrangement of TCR-γ, *ASP* Asymptomatic polyclonal rearrangement of TCR-γ, *TCR-γ* T-cell antigen receptor γ-chain, *HCs* Healthy controls, *ND* Not determined, *NA* Not available, *PBMCs* Peripheral blood mononuclear cells

### Description of whole-genome sRNA-sequencing data from each group

sRNA sequencing generated a total of 149,424,024 reads using the Illumina MiSeq platform. After the exclusion of low-quality reads, 124,171,839 clean reads were mapped to selected genic regions. The filtered sRNA reads for each sample were aligned to the human genome sequence dataset and are depicted in Table 1. The MPS approach yielded 381 sRNA molecules consisting of 307 and 74 known and novel sRNAs, respectively. Of the 307 known sRNA, 203 were miRNA, 52 tRNA, 25 snoRNA, 12 scRNA, 5 scRNA_pseudogene, 7 snRNA, and 3 piRNA (Table S1). The analysis also identified 234 mature miRNA, with 22 having expression greater than a two-fold change. Of the 74 novel genes, 8 were novel miRNA, 18 snoRNA, and 48 were unknown (Table S2).

### Expression profiling of the known sRNAs

**Fig. 1:**
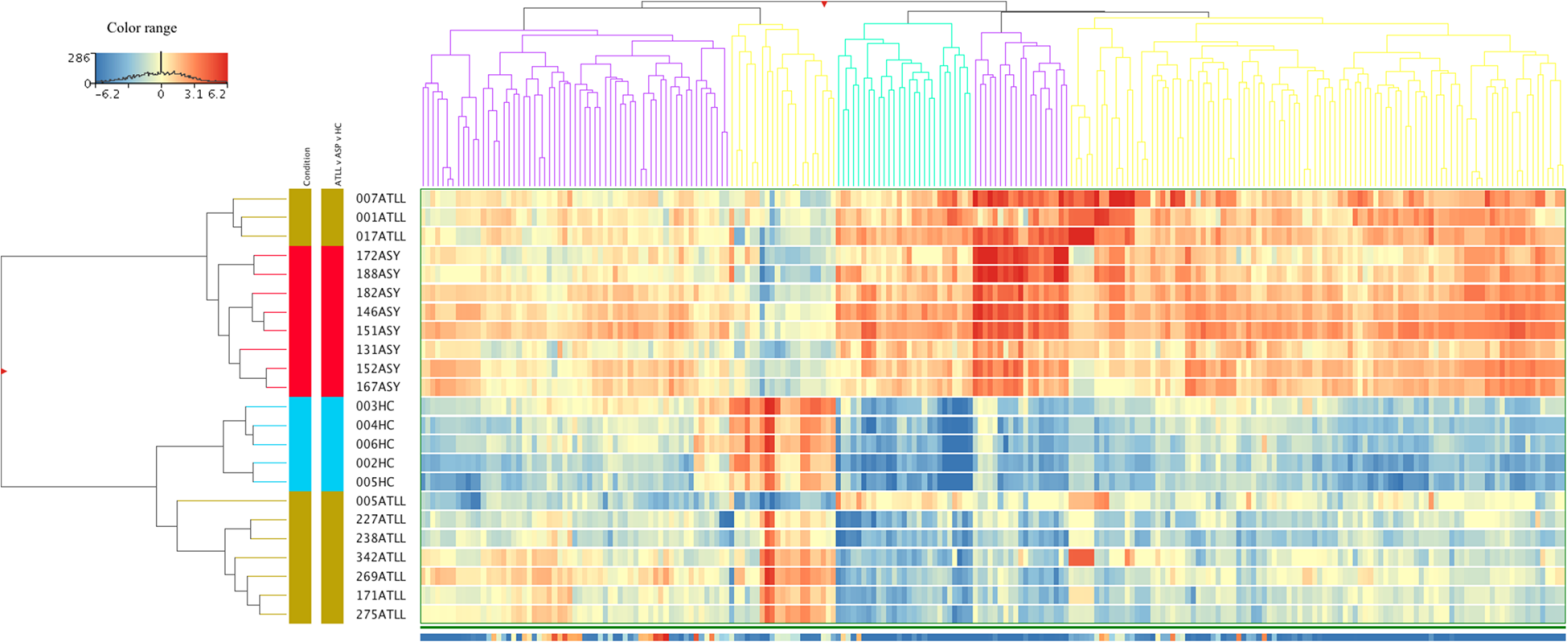
Unsupervised hierarchical clustering showing 226 differentially expressed sRNAs among ATLL patients (golden colors), ASP subjects (red colors), and health carriers (blue colors). The sample-clustering tree is displayed to the left and the sRNA clustering tree is above forming 5 clusters as indicated by colors. The color scale at the top indicates the relative expression levels of sRNA across all samples: red color indicates overexpressed sRNAs and blue color; blue indicates under expressed sRNAs. Each column represents one known sRNA and each row represents one sample. ATLL; Adult T cell lymphoma/leukemia, ASP; asymptomatic carriers with polyclonal T cell receptor γ gene rearrangement; HC, healthy control

Of the 307 known sRNAs identified in this study, 226 sRNAs were differentially expressed among the three groups (p (corr) < 0.05), 184 and 26 were up and downregulated, respectively, (in both ATLL and ASP groups) in comparison with the HC group (Table S3). The expression profiles of the 226 above-identified sRNA were included in the subsequent analysis. Unsupervised cluster analysis based on the Euclidean distance was used to visualize their expression profiles. The results revealed a differential pattern of expression with five main clusters in which acute type-ATLL, other clinical type-ATLL, ASP, and HC groups were segregated (Fig. 1). Interestingly, one patient (005ATLL) classified as chronic-type ATLL at the time of sample collection, progressed to aggressive acute phase after 2 years and died with leukemia 5 months after the onset of the acute phase. Another observation of this analysis is that the sRNA profiles of patients with non-acute ATLL were even more similar to the ASP group in some instances. The first cluster was composed of 61 sRNAs with lower expression levels than the HC group. In the second cluster, a differential expression pattern was observed with 21 upregulated genes in HC and ATLL patients. The 3rd, 4th, and 5th clusters revealed 27, 19 (mostly snoRNA), and 98 genes, respectively, with an expression pattern of positive regulation in ASP and nonacute ATLL –type patients.

In further analysis, we aimed to identify differentially known and novel expressed sRNAs using one-way ANOVA and pairwise contrasts, with the Benjamini-Hochberg [21] correction (*p* < 0.05) on the samples in the three groups. In the ATLL group, we found different expression levels of 41 of the known sRNAs from the ASP and HC groups, of which 15 and 26 were upregulated and downregulated sRNAs, respectively (Table S3). Next, after correction for multiple testing (Benjamini-Hochberg) and application of a moderated t-Test p cutoff = 0.05, we compared the expression profiles of the known sRNA between the ATLL and HC subjects. The results indicated that 114 of the 226 known sRNAs remained significantly differentially expressed, of which 100 were upregulated and 14 were downregulated. The upregulated sRNA in the ATLL group included 70 miRNA, 2 piRNA, 8 snoRNA, 7 snRNA, and 13 tRNA. On the other hand, the downregulated entities include 13 miRNA and only one scRNA_pseudogene. hsa-mir-150, trna65, has-let-7b, and hsa-mir-331 were of the highly expressed sRNAs (p (corr) < 0.05) (Table S4). The four most strongly downregulated sRNAs in the PBMCs of the ATLL group were hsa-mir-19a, hsa-mir-19b-1, hsa-mir-19b-2, hsa-mir-7-3, and hsa-mir-7-2, among which hsa-mir-19a had greater than 27 fold down.

Then, we used the same strategy to compare the expression profiles of the known sRNA of the ASP and HC groups. The analysis revealed 216 of the 226 known genes satisfying corrected *p*-value cutoff < 0.05, and most (90.3%) were upregulated in the ASP subjects (Table S5). The 21 downregulated sRNAs included 20 miRNA and only one tRNA, namely trna9. The upregulated entities in the ASP group include 121 miRNA, 3 piRNA, 9 scRNA, 2 scRNA_pseudogene, 23 snoRNA, 7 snRNA, and 30 tRNA. The five most strongly upregulated sRNAs in the ASP group were hsa-mir-150, hsa-mir-146a, hsa-let-7e, hsa-mir-342, and hsa-mir-28. The five most significantly downregulated sRNAs in PBMCs of the ASP group were hsa-mir-486, hsa-mir-486-2, hsa-mir-183, hsa-mir-144, and 451a.

Finally, we evaluated the differentially expressed genes between ATLL and ASP. The data showed that of the 226 known sRNAs, 149 remained differently expressed and 140 were downregulated (Table S6). All nine upregulated genes in the ATLL group were miRNAs, of which hsa-mir-451a and hsa-mir-183 were the top two upregulated entities. The 140 downregulated sRNA included 100 miRNA, 1 piRNA, 8 scRNA, 2 scRNA_pseudogene, 21 snoRNA, 7 snRNA, and 17 tRNA. The five most strongly downregulated sRNAs in the PBMCs of the ATLL group when compared with the ASP subjects (p (corr) < 0.05) were hsa-mir-26a-2, hsa-mir-26a-1, trna15, hsa-mir-4772. Of these, hsa-mir-26a-2 was the most downregulated sRNA in both Benjamini–Hochberg FDR (0.001) and ranking of fold change (− 10).

**Fig. 2:**
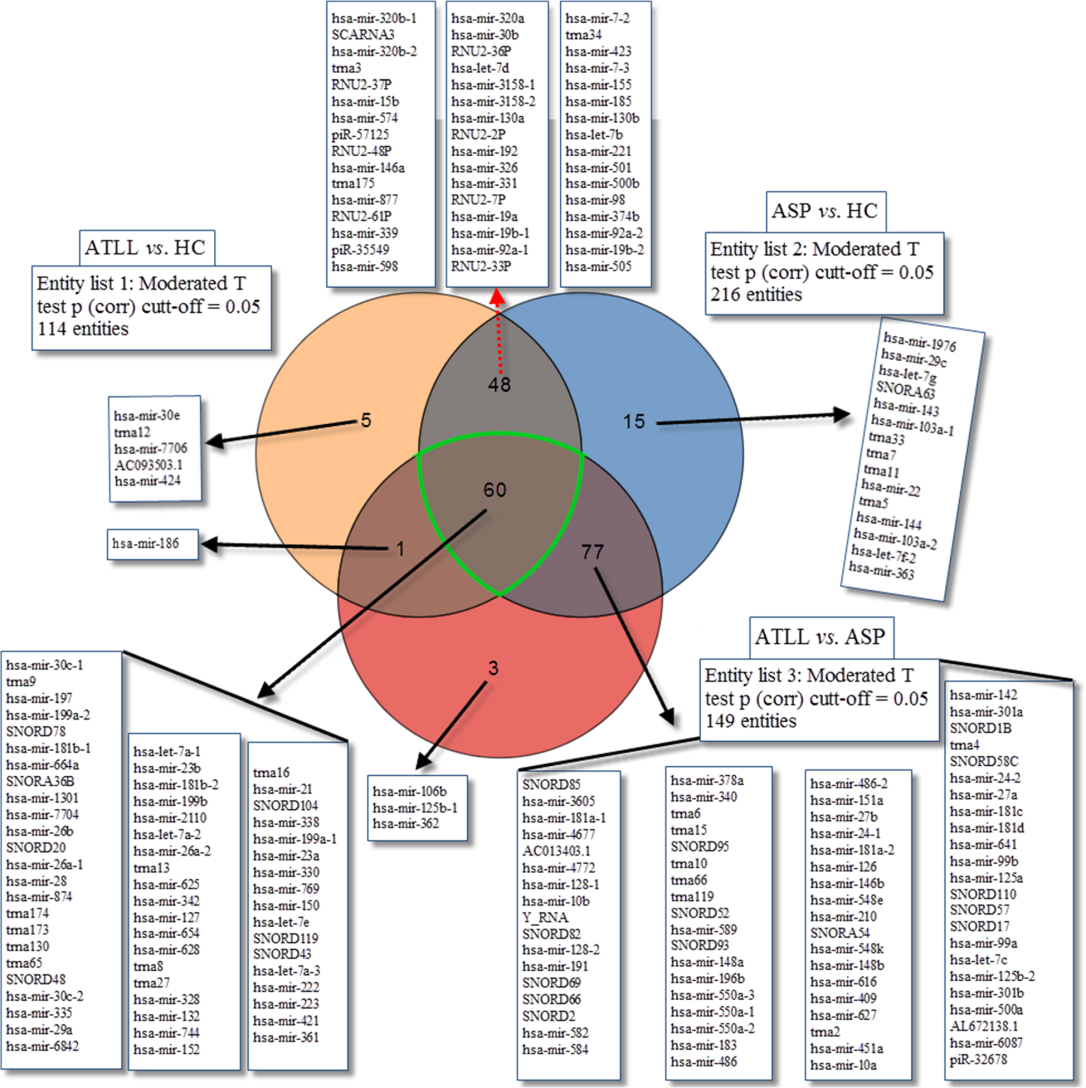
Comparisons of significantly dysregulated known sRNAs in peripheral blood mononuclear cells of HTLV-1 associated ATLL patients versus ASP and HCs. The Venn diagram indicates the number of sRNAs differentially expressed in entity list 1 (ATL vs. HC), entity list 2 (ASP vs. HC) and entity list 3 (ATL vs. ASP). Expression levels of these sRNAs are detailed in supplementary Tables S1, S2 and S3

A Venn diagram representation of the number of all sRNAs overlapping in different comparisons among the three groups is depicted in Fig. 2. The results showed 60 sRNAs were common among the 3 groups, while 5 sRNAs, 15 sRNAs, and 3 sRNAs were specific in entity 1 (ATLL vs. HC), entity 2 (ASP vs. HC), and entity 3 (ATLL vs. ASP), respectively.

### Expression profiling of the novel sRNAs

Sequencing of the library and subsequent analysis of the three groups revealed 74 new sRNAs, of which 51 were differentially expressed (28 unknown, 16 snoRNAs, and 7 miRNAs). After correction for multiple testing, the data showed that 26 of the 51 novel sRNAs remained significantly dysregulated in the ATLL vs. HC group; 17 and 9 sRNAs were upregulated and downregulated, respectively. The reads of these 26 novel genes were annotated as 14 unknown novel genes, 3 miRNAs, and 8 snoRNA (Table S7). Comparison of data after correction for multiple testing between the ATLL and ASP groups demonstrated that 27 novel sRNAs were strongly dysregulated; 6 and 21 sRNAs were upregulated and downregulated, respectively. The 27 new sRNA include 14 unknown novel genes, 9 snoRNA, and 4 miRNA (Table S8). All four novel sRNA that displayed high expression abundance were unknown sRNAs. Of the five top strongly downregulated sRNAs, three were unknown and there was one miRNA and one snoRNA. Finally, the same analysis strategy was used to compare the ASP and HC groups. The results revealed significant dysregulation of 48 genes in the ASP subjects when compared to the HC group and were annotated as 27 unknown novel genes, 6 miRNAs, and 15 snoRNA (data not shown). Among these significantly deregulated novel genes, 34 and 14 sRNAs displayed high and low expression abundance, respectively. The four top strongly upregulated novel sRNAs were annotated as two miRNA and snoRNA each, whereas 100% of the novel entities that displayed low expression abundance were annotated as unknown genes.

### Expression profiling of the known mature miRNAs

To reduce bias depending on the coverage, we considered only known mature miRNAs covered by at least 20 reads. A total of 234 mature miRNAs fulfilled the criteria, 164 of which were significantly differentially expressed in the PBMCs of the three groups. For known mature miRNAs after correction for multiple testing, 49 of the 164 active miRNAs remained significantly dysregulated (p (corr) < 0.001) (Table S9). The top 10 most highly expressed miRNAs among all samples were hsa-miR-199a-3p, hsa-miR-26a-5p, hsa-miR-199b-3p, hsa-miR-150-5p, hsa-let-7d-3p, hsa-miR-155-5p, hsa-miR-26b-5p, hsa-miR-222-3p, hsa-miR-181b-5p, and hsa-miR-30e-3p. The individual analysis between the ATLL and HC subjects displayed nine entities of 55 satisfying the corrected *p*-value (Benjamin-Hochberg FDR) cutoff < 0.001; eight upregulated and one downregulated miRNAs (Table S10). Among the nine miRNAs, the uniquely downregulated hsa-mir-19a-3p and the upregulated hsa-let-7d-3p, followed by 199b-3p, miR-331-3p, and hsa-mir-199a-3p, exhibited the most prominent differences. In the analysis of the miRNAs of the ATLL group vs. the ASP group, three of the 55 miRNAs, hsa-mir-146b-5p, hsa-mir-26a-5p (Entrez 407,015), and hsa-mir-26a-5p (Entrez 407,016) showed considerably low expression levels (p (corr) < 0.001). Finally, the differential regulation of miRNAs between the ASP and HC groups was computed and revealed 48 of the 49 active miRNAs displaying high (*n* = 46) and low (*n* = 2) expression abundance. Hsa-mir-150-5p, hsa-mir-146a-5p, hsa-mir-342a-3p, hsa-mir-181a-2-3p, and hsa-mir- 23a-3p were annotated as the five top considerably upregulated genes. Hsa-mir-451a and hsa-mir-19a-3p were the unique miRNAs that displayed low expression abundance (Table S11).

**Fig. 3:**
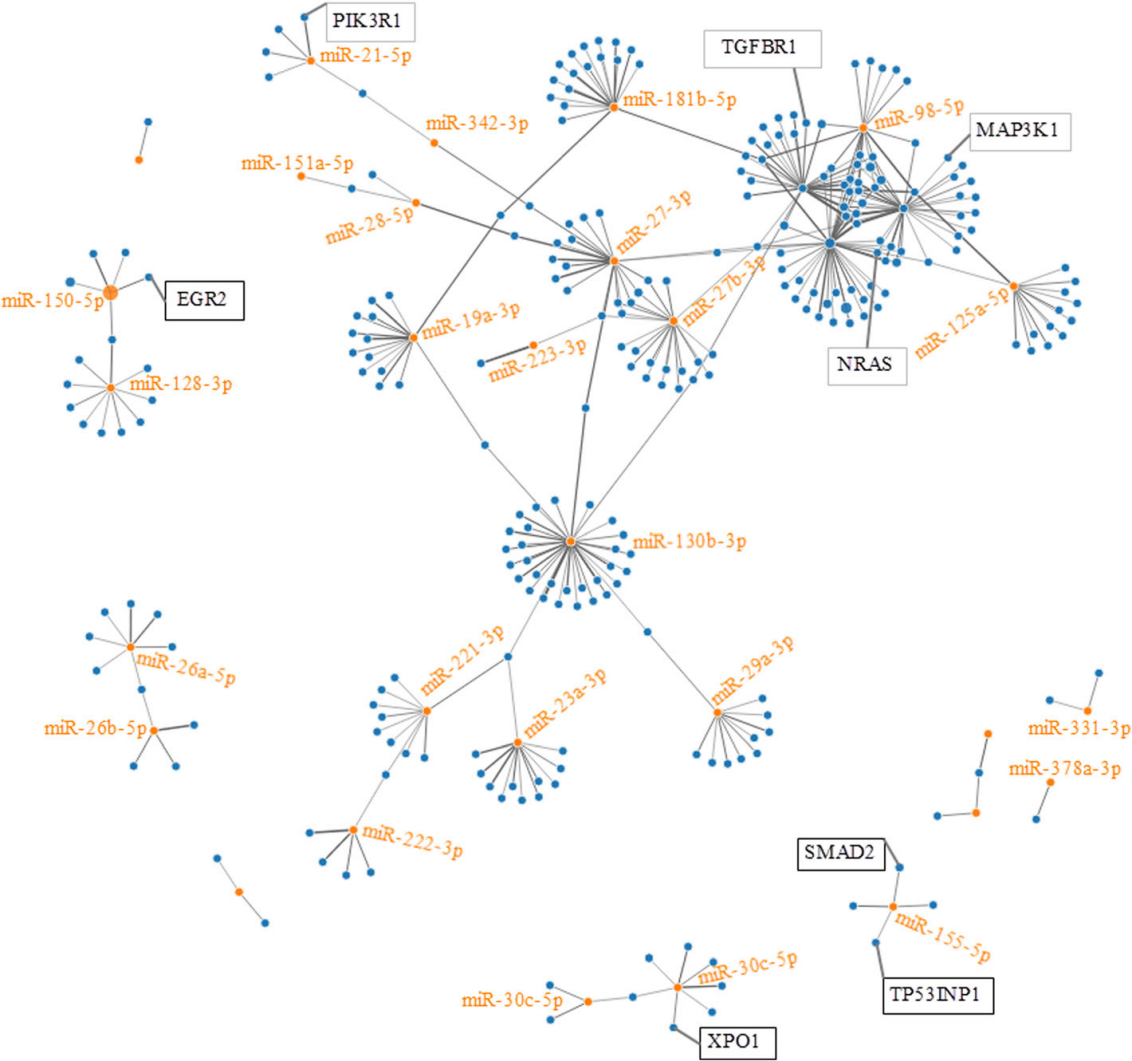
Interaction network between top 10 dysregulated miRNAs and their target genes. Blue circles represent mRNAs, while orange circles represent miRNAs. The more connections between miRNAs and genes, the more links within the network

We then used the mirWalk algorithm to generate a network and hence determine and rank the miRNA-mRNA putative target interaction. As shown in the interaction map (Fig. 3), the blue circles are ATLL-relevant mRNAs targeted individually by, for example, miR-155-5p, miR-150-5p, miR-30c-5p, and miR-98-5p. ATLL-specific mRNAs were targeted, for instance, by miR-26a-5p, miR-26b-5p, miR-331-3p, and miR-378a-3p.

### Validation by qRT-PCR assay

**Fig. 4:**
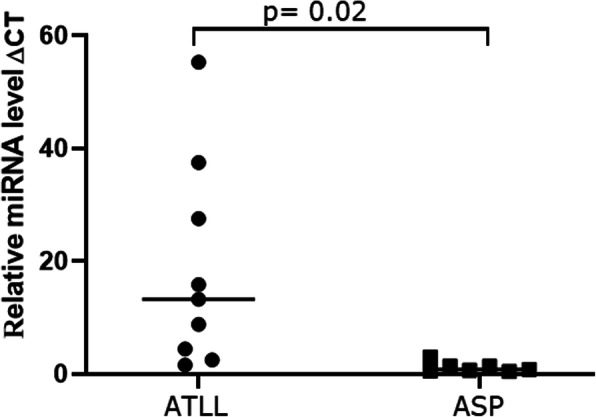
Relative expression levels (ΔCT) of hsa-miR-451a in peripheral blood mononuclear cells of HTLV-1-associated ATLL patients versus asymptomatic carriers with polyclonal T cell receptor γ gene rearrangement

To further validate the screen of miRNA expression with an independent qRT-PCR method using the samples from a total of nine ATLL patients used for sequencing, we chose two of the most differentially regulated miRNAs, hsa-mir-451a and hsa-mir-144, among ATLL-relevant miRNAs. As shown in Fig. 4, the expression levels of the selected mir-451a were significantly downregulated in ATLL patients compared with the ASP group. The level of hsa-mir-144 could not be accurately quantified in most samples because of its low abundance or absence.

### Target genes analysis

By applying increasingly stringent statistical methods, the current study identified 49 non-redundant mature miRNAs that were significantly dysregulated in the PBMCs of the HC and ASP groups when compared with the ATLL patients (p (corr) < 0.001). Since miRNAs regulate target gene expression at the post-transcriptional level, we predicted the target genes of dysregulated miRNAs using the miRWalk 3.0 algorithm, which provides a comprehensive list of putative and validated mRNAs for a given miRNA by combining results from 12 established target prediction algorithms for the identification of putative miRNA targets. A total of 404 non-redundant genes were predicted as putative target genes of the dysregulated miRNAs (Table S12). The total numbers of high confidence mRNA hits generated by hsa-let-7b-5p, hsa-let-7a-5p, hsa-miR-130b-3p, hsa-let-7d-5p, and hsa-miR-27b-3p were 62, 53, 34, 33, and 51, respectively. Our manual approach for the identification of ATLL-relevant mRNAs targeted by the selected miRNAs yielded XPO1, TGFBR1, EGR2, NRAS, SMAD2, PIK3R1, E2F2, TP53INP1, and MAP 3 K1 (supplementary Figure 1).

### GO/pathway enrichment analysis of target genes

To understand the role of miRNAs in the progression of ATLL in HTLV-1 infected patients, GO and KEGG pathway enrichment analysis were carried out. The top GO terms at the MF level were; cytokine-mediated signaling pathways and transcription initiation from RNA polymerase II promoter. The top GO terms at the CC level were; nuclear chromatin and promyelocytic leukemia body. The top GO terms at the MF level were; RNA polymerase II proximal promoter sequence-specific DNA binding and transcription_factor_binding. GO analysis results showed that upregulated DEGs were significantly enriched in binding, protein binding, and organic cyclic compound binding at the MF level. The KEGG analysis revealed that the upregulated differentially expressed miRNAs were mostly enriched in various pathways, iwhere human T-cell leukemia virus 1 infection and cancer were the most significantly enriched pathways (FDR corrected *p*-value of ≤0.05). Of note, cancer pathways are a combination of several pathways, including pathways reported to play a key functional role in ATLL pathology (TGF-β, Wnt, p53, apoptosis, and MAPK signaling), indicating that several vital processes might be regulated by these miRNAs (supplementary Figure 2).

## Discussion

In the present study, a genome-wide approach was used for the investigation of miRNA profiles in the PBMCs from ATLL patients. The overall analyses revealed 226 and 51 differentially expressed known and novel sRNA, respectively. In addition, we also detected 49 known mature miRNAs with significantly different expression among all three groups (ATLL, ASP, and HC). All these miRNAs, except hsa-mir-451a and hsa-mir-183 were significantly downregulated in ATLL patients. Hsa-miR-150-5p and 146a-5p were among the top dysregulated miRNAs in ATLL patients vs. ASP and HC groups. Many studies have demonstrated different expression patterns of miRNAs in ATLL patients and HTLV-1-transformed cells, of these; miR-155, miR-146a, miR-150, and miR-223 were upregulated and miR-31 and miR124a downregulated [29, 30, 32, 44]. Consistent with these previous reports, the present study revealed that several miRNAs were associated with leukemia transformation within the ATLL group, supporting the potential involvement of miRNAs in the pathogenesis of HTLV-1-associated ATLL. For instance, the downregulation of miR-146a, miR-155, miR-150, miR-22, and miR-130b was reported to affect cellular proliferation [45–48].

To determine the biological significance of the mature deregulated miRNAs in ATLL, in silico target prediction was performed and resulted in a total of 270 experimentally validated target mRNAs. Identification of ATLL-relevant mRNAs targeted by the selected miRNAs yielded in mRNAs CREB, TGFBR1, EGR2, NRAS, SMAD2, PIK3R1, E2F2, TP53INP1, and MAP3K1. The qRT-PCR expression preferences of the selected differentially expressed miRNAs agreed with the results from MPS. The majority of the enriched GO terms at the MF level were potentially involved in cellular binding, which is considered a basic step in the regulation process. It has been known that the GO terms enriched binding is consistent with the knowledge that growth and metastatic progression of solid tumors and hematological malignancies are controlled by multiple signals and targeting multiple molecules [49, 50]. Profiling of sRNAs has been acknowledged as a potential utility in unraveling regulation of biological pathways [51, 52] and in the discovery of disease-related biomarkers [53–56]. The vast majority of expression studies of sRNAs in HTLV-1-infection either focused on a few key entities as potential regulators of T cell transformation and pathogenicity or used the qRT-PCR and microarray analysis to investigate the dysregulation of miRNA in HTLV-1-infected cell lines and ATLL transformed cells [29–31, 57]. Among the reported miRNAs, miR-155, miR-146a, miR-150, and miR-223 were upregulated while miR-31 and miR124a down-regulated [29, 30, 32]. The expression profiles of many mature miRNAs identified in this study are consistent with those of previously published studies and have known responses in various cellular processes, including cell proliferation, differentiation, and apoptosis. For instance, in agreement with a previous study by Pichler et al. [29], which examined the expression of miRNAs in HTLV-1-transformed cells, our findings from clinical samples demonstrated the upregulation of miR-21, miR-146a, miR-155, and miR-223 in PBMCs of ATLL associated HTLV-1 infected subjects. Of note, the three miRs (miR-21, miR-146a, miR-155), together with miR- 27a reported in our study (Table S10), were found upregulated in Epstein-Barr virus-infected B-cells during latency III, the viral growth program that induces B-cell proliferation [58].

Surprisingly, some of the previously published reports revealed little overlap between identified miRNAs expression data in ATLL-infected HTLV-1 patients [29, 30]. This discrepancy could arise from experimental differences and different cellular compartments [59]. It is also possible that the small sample size and genetic variation of human populations could have contributed to the inconsistent results of miRNA differences in ATLL. The current work compared HTLV-associated ATLL in asymptomatic and healthy subjects, resulting in a diverse number of sRNAs that differed among the three groups. As evident from our bioinformatics approach, we found that TGF-β, Wnt, PI3K, p53, apoptosis, and MAPK signaling pathways, which have been implicated in ATLL pathogenesis [59–63], were significantly targeted by the active miRNAs modulated in the ATLL patients. The TGF-β signaling pathway plays a crucial role in cellular homeostasis, and that inactivation of this pathway has been demonstrated in HTLV-1–induced ATLL through the action of the viral oncoprotein tax [64, 65]. Of the known miRNAs upregulated by TGF-β signaling and detected in this study are hsa-miR-155-5p [66], hsa-miR-181b-5p [67, 68], hsa-miR-21-5p [69, 70], hsa-miR-23a-3p [71], and hsa-miR-27b-3p [72]. In addition to the distinct pattern of mature miRNA expression in the PBMCs from ATLL patients when compared with the ASP and HCs subjects, we found that miR-451a was significantly downregulated in ATLL patients. Our identification of an miR-451a signature in the PBMCs from ATLL patients associated with HTLV-1 infection is a novel finding and has the potential to be a selective biomarker for ATLL development. It has been reported that miR-451 is downregulated in cytogenetically normal acute myeloid leukemia patients [73], and miR-451 functions as either a tumor suppressor [74, 75] or an oncogene [76, 77] in human glioma cells. A previous study provided evidence that expression of miR-451 in intracellular Notch1 (ICN1)-expressing bone marrow completely blocked the initiation of T cell acute lymphoblastic leukemia in recipient mice leaving normal T cell development and the generation of nonmalignant ICN1-overexpressing cells intact, indicating that reduced expression of these tumor suppressor genes was required for transformation [78]. Cheng et al. [79] reported elevated expression of ICN1 in cell lines infected with HTLV-1 and that repression of cell proliferation and tumor formation in vitro and in vivo can be achieved by Knocking down ICN1 in ATL cells. The same study proposed that HTLV-1 promotes of growth of ATL cells by regulating Notch signaling through a posttranslational event that involves interactions of the viral tax protein with the Notch intracellular domain and the recombination signal binding protein immunoglobulin Jκ (RBP-jκ). Thus, it is reasonable to speculate that the repression of miR-451 may be essential for ICN1-induced oncogenesis in HTLV-1 associated ATLL. Another hypothesis suggested by the results of Ansari et al. [79] in glioblastoma cells is that the miR-451 level expression is dependent on glucose metabolism and is suppressed after AMP-activated protein kinase (AMPK) activation during glucose deprivation [77, 80, 81]. It is tempting to speculate that a similar mechanism may hold true for HTLV-1 associated ATLL since both AMPK and glucose levels have been implicated in ATLL progression. For instance, the reactivation of HTLV-1 from latency is severely limited in the absence of glucose [82] however, glucose deprivation can cause upregulation of AMPK through downregulating miR-451. Consistent with this hypothesis, a recent study by Aikawa et al. [82] reported higher levels of AMPK in acute and chronic ATL than in asymptomatic HTLV-1 carriers and healthy donors. Although attractive, these hypotheses have not been proven and await experimental testing.

Our results also revealed 51 significantly dysregulated novel sRNAs, including 28 unknown, 16 snoRNAs, and 7 miRNAs that meet the miRNA classifications criteria. Validation and future analysis will certainly be required to address the regulatory mechanisms and biological effects of these small molecules. We do not understand the biological and clinical implications of these novel molecules; however, it is anticipated that a better understanding of these entities will be fertile ground for future experiments.

Our pilot study has several limitations, particularly its retrospective design with a small number of study patients. Thus, future studies with larger sample sizes are needed to validate the findings of this pilot study. Despite the above limitations, the current pilot study adds further support to previous studies by providing a link between HTLV-1 associated ATLL and sRNA expression patterns. The aberrant expression of sRNAs described here might open doors for future studies aimed at investigating their mechanistic roles and assessing their potential clinical roles as predictors, and/or therapeutic targets of HTLV-1 associated ATLL.

## Conclusions

Our pilot study identified specific sRNA signatures for HTLV-1 associated ATLL that could potentially be used as biomarkers to detect ATLL at early stage. miR-451 may offer a good therapeutic target in ATLL via the AMPK/ Notch signaling pathway.

## Supplementary Information

**Additional file 1: Table S1.** List of known sRNAs differentially expressed in ATLL patients vs asympotomatic HTLV-1 infected subjects with polyclonal T cell expansion (ASP) and healthy control (HC)subjects.

**Additional file 2: Table S2.** A full list of the 74 novel small RNAs identified in this study.

**Additional file 3: Table S3.** List of the 226 known sRNAs differentially expressed among ATLL, ASP, and healthy controls.

**Additional file 4: Table S4.** List of the 114 known sRNAs differentially expressed between the ATLL and HC group.

**Additional file 5: Table S5.** List of the 216 known smallRNAs differentially expressed between ASP and HC group.

**Additional file 6: Table S6.** List of the 149 known smallRNAs differentially expressed between ATLL patients and ASP subjects.

**Additional file 7: Table S7.** List of the 26 novel sRNAs differentially expressed between ATLL and HC group.

**Additional file 8: Table S8.** List of the 27 novel sRNAs differentially expressed between ATLL patients and ASP subjects.

**Additional file 9: Table S9.** List of the 49 mature miRNAs differentially expressed in ASP and HC subjects compared to ATLLpatients.

**Additional file 10: Table S10.** List of the nine mature miRNAs differentially expressed between the ATLL patients and HC group.

**Additional file 11: Table S11.** List of the 48 mature miRNAs differentially expressed between the ATLL patients and HC group.

**Additional file 12: Table S12.** Putative target genes of differential miRNAs.

**Additional file 13: Figure S1.** Pathways in HTLV-1 infection (from KEGG). This pathway was significantly enriched in the KEGG analysis. Objects selected with red color are the acting locations by mapped targeted genes.

**Additional file 14: Figure S2.** Pathways in cancer (from KEGG). This pathway was significantly enriched in the KEGG analysis. Objects selected with red color are the acting locations by mapped targeted genes.

## Data Availability

All sequence data described here are available in the online Zenodo repository (10.5281/zenodo.3909819 and 10.5281/zenodo.1181925).

## Abbreviations

sRNA: Small RNA
miRNA: MicroRNA
ncRNA: Non-coding RNA
tRNA: Transfer RNA
rRNA: Ribosomal RNA
snRNA: Small nuclear RNA
snoRNA: Small nucleolar RNA
scRNA: Small cytoplasmic RNA
PBMC: Peripheral blood mononuclear cell
HTLV-I: Human T-lymphotropic virus type I
ASM: Asymptomatic monoclonal
ASP: Asymptomatic polyclonal
ATLL: Adult T-cell leukemia/lymphoma
