# Supplementary figures and images for: Global expression of noncoding RNome reveals dysregulation of small RNAs in patients with HTLV-1–associated adult T-cell leukemia: a pilot study
Source: Infect Agent Cancer. 2021 Jan 9;16:4. doi: 10.1186/s13027-020-00343-2 (PMC7797118; doi:10.1186/s13027-020-00343-2)

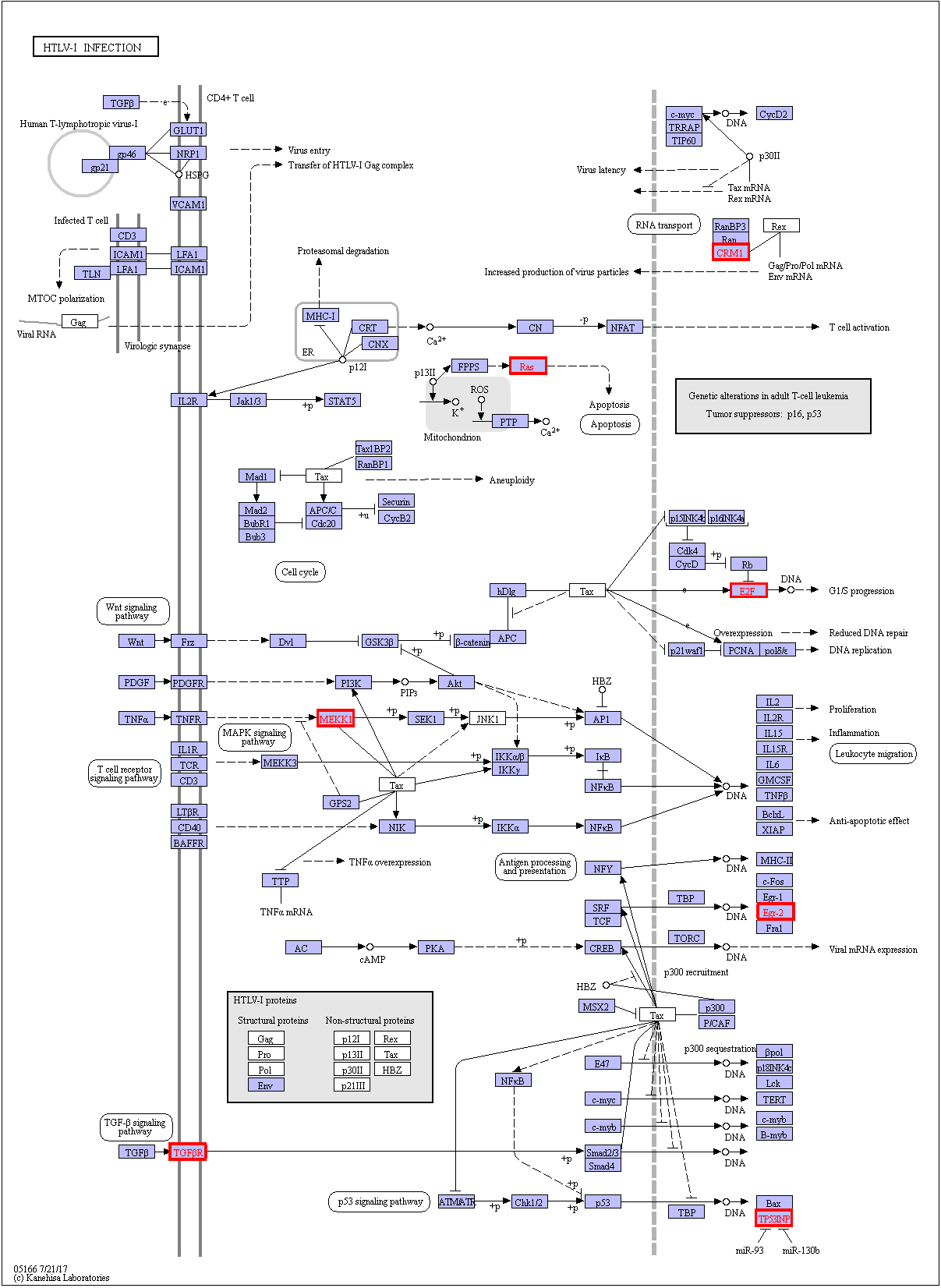

Supplement: Supplementary file 13 — Additional file 13: Figure S1. Pathways in HTLV-1 infection (from KEGG). This pathway was significantly enriched in the KEGG analysis. Objects selected with red color are the acting locations by mapped targeted genes. [file 13027_2020_343_MOESM13_ESM.tif]

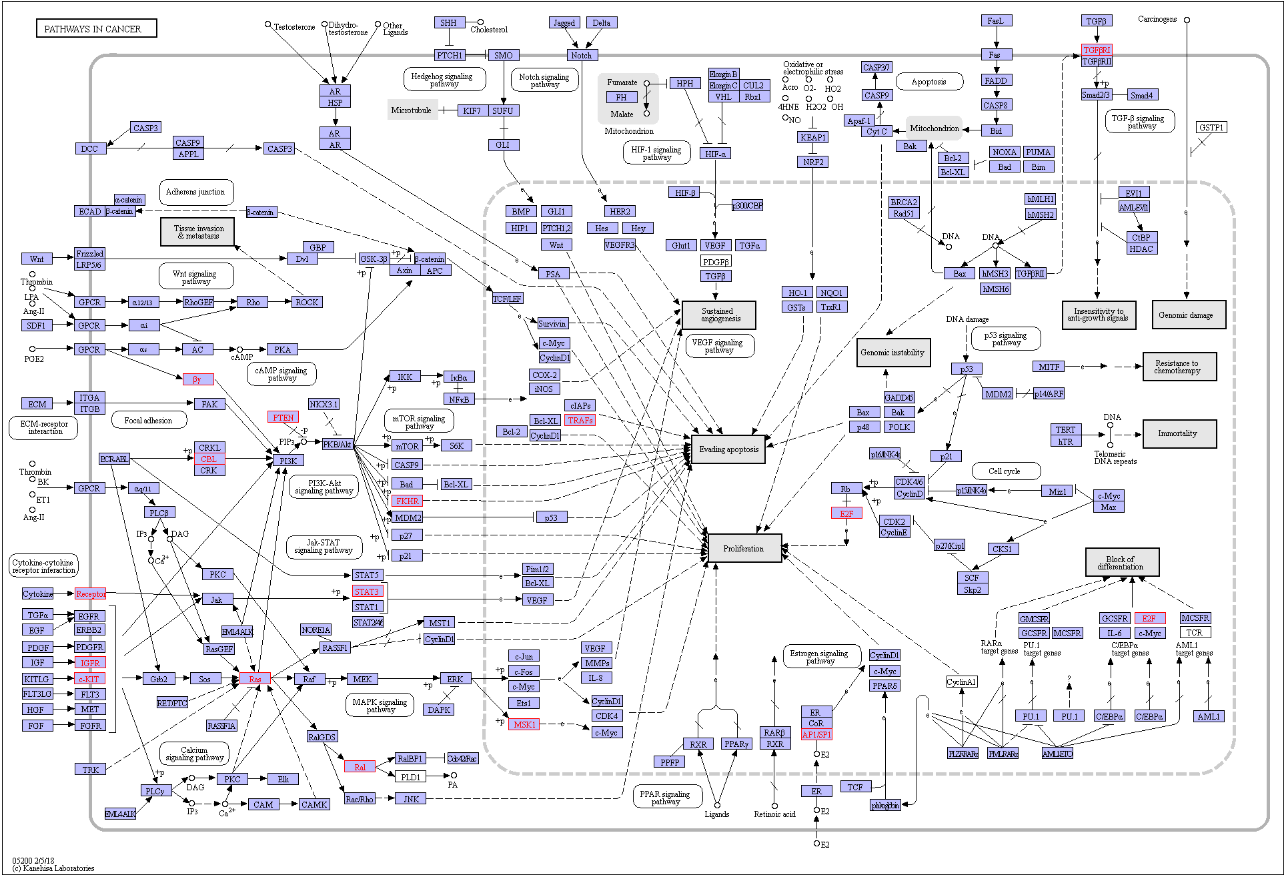

Supplement: Supplementary file 14 — Additional file 14: Figure S2. Pathways in cancer (from KEGG). This pathway was significantly enriched in the KEGG analysis. Objects selected with red color are the acting locations by mapped targeted genes. [file 13027_2020_343_MOESM14_ESM.tif]
